# Employee Perceptions of Workplace Health Promotion Programs: Comparison of a Tailored, Semi-Tailored, and Standardized Approach

**DOI:** 10.3390/ijerph15050881

**Published:** 2018-04-28

**Authors:** Tamara D. Street, Sarah J. Lacey

**Affiliations:** Wesley Medical Research, Auchenflower QLD 4066, Australia; slacey@wesleyresearch.com.au

**Keywords:** affect, employee perceptions, occupational health, program evaluation, utility, workplace health promotion, workplace wellness

## Abstract

In the design of workplace health promotion programs (WHPPs), employee perceptions represent an integral variable which is predicted to translate into rate of user engagement (i.e., participation) and program loyalty. This study evaluated employee perceptions of three workplace health programs promoting nutritional consumption and physical activity. Programs included: (1) an individually tailored consultation with an exercise physiologist and dietitian; (2) a semi-tailored 12-week SMS health message program; and (3) a standardized group workshop delivered by an expert. Participating employees from a transport company completed program evaluation surveys rating the overall program, affect, and utility of: consultations (*n* = 19); SMS program (*n* = 234); and workshops (*n* = 86). Overall, participants’ affect and utility evaluations were positive for all programs, with the greatest satisfaction being reported in the tailored individual consultation and standardized group workshop conditions. Furthermore, mode of delivery and the physical presence of an expert health practitioner was more influential than the degree to which the information was tailored to the individual. Thus, the synergy in ratings between individually tailored consultations and standardized group workshops indicates that low-cost delivery health programs may be as appealing to employees as tailored, and comparatively high-cost, program options.

## 1. Introduction

Workplace health promotion programs (WHPPs) have become an increasingly popular means of promoting positive health behaviors in employees that are mutually beneficial to employers and employees [1,2,3]. Such programs can improve the overall health of the individual [4,5,6,7], increase physical activity [8,9], lead to small improvements in healthy weight status [6,10], have positive effects on dietary behaviors [3,11] and improve employee productivity [12,13,14]. Whilst there is overall support for the effectiveness of WHPPs, the reported extent to which such programs achieve lasting changes in behavior varies [10,15,16]. This variation in reported effectiveness is expected given the diversity in the design and quality of programs offered.

Scientific reviews have shown that the design of successful WHPPs is a complex process and programs are most effective when they are: (1) based on scientifically valid constructs [17,18,19,20,21,22]; (2) tailored to the meet the needs of individuals [6,10,21,23,24,25,26]; and (3) perceived as both useful and enjoyable by participants [7,9,27,28,29,30]. Despite an abundance of practitioner research focused on validation of scientific constructs (e.g., the application of social cognitive theory [17,31] or behavioral strategies [21,32]), and objective outcomes of WHPP participation (e.g., changes in Body Mass Index (BMI) and nutritional consumption [8,10,11,33]); few studies have focused on employee perceptions of WHPPs associated with participation and engagement. This omission in the literature is surprising as employee participation and evaluations are associated with engagement in WHPPs [34,35,36]. Therefore, employee perceptions of WHPPs are of high relevance and importance in informing program design and refinement of delivery.

In practice, WHPPs are often designed and delivered by workplace health and safety (WH&S) professionals with limited specialized training in health intervention design and evaluation, or access to current scientific publications. As a result, practitioners tend to employ a “one-size-fits-all” approach (e.g., offering of a standardized boot camp), frequently with cost rather than engagement and outcomes being the primary driver of program selection [5,23,24,29,37]. Hence, it is typically the practitioner’s opinions about program design, mode of delivery, and content that are considered during the process of program definition and selection, while the perspective of employees’—whom are subsequently asked to choose whether or not to participate—remain overlooked [7,17,29]. This “one-size-fits-all” approach often fails to attract considerable employee participation and engagement, especially among the individuals who would benefit most from the program but are least likely to volunteer, particularly when grouped with colleagues who are already engaging in healthy lifestyle behaviors. In turn, such a practitioner-driven program design approach can result in low participation and/or high rates of attrition [10,33,38].

Of the research investigating participation and engagement, Crutzen et al. [20,30] developed an evaluation strategy to measure user perceptions of health promotion interventions based on the Technology Acceptance Model [39]. In addition to overall impressions of the program, the authors derived two key constructs of program user perceptions. The constructs were characterized as ‘affective user perceptions’ and ‘cognitive user perceptions of utility’. More specifically, Crutzen et al. [30] posited that sustained participant engagement in a health promotion program can be achieved through positive affect (i.e., emotional responses related to: enjoyment; interest; and support) and high perceptions of program utility (i.e., cognitive evaluations related to: ease of use; outcomes; and program value). In the design and evaluation of WHPPs, participant perceptions of affect and utility predict an individual’s participation and engagement [30]. A positive affect is likely to increase interest in the program and result in increased time participating [40]; whilst positive perceptions of utility (i.e., user experience) lead to increased participant loyalty and dedication to the program [41].

Given the important contribution of employee perceptions to the effectiveness of any WHPP, and the paucity of scientific literature that considers these constructs, further investigation is warranted to validate the measurement, application, and evaluation of employee perceptions of WHPPs. In particular, it is important to investigate whether any synergy exists between the often-selected “one-size-fits-all” standardized, cost-effective WHPP design and that of a more tailored but higher-cost approach recommended in the literature. At the time of publication, the authors were unable to identify any study that provided a comparison of employee perceptions of WHPPs categorized by the degree of program tailoring or mode of delivery, and which could be associated with the comparative cost of program implementation. Thus, the purpose of this study was to compare employee perceptions of the overall program offered, together with perceptions of program affect and utility across WHPPs that differed in their levels of tailoring and mode of delivery.

The three WHPPs included in the study were designed by a single research team with expertise in health behavior change, psychology, exercise physiology, and dietetics. All programs aimed to increase healthy lifestyle behaviors including physical activity and nutritional consumption. The programs comprised:

(1) A fully tailored one-on-one health consultation with a registered dietitian and exercise physiologist;
(2) A semi-tailored SMS health messages program with tailoring based on employees’ reported readiness to change exercise and eating behaviors; and
(3) A standardized group health workshop presentation delivered by a registered dietitian and exercise physiologist.

It was hypothesized that the participant ratings of the overall program, as well as participant ratings of affect and utility, would be highest in the individual tailored condition, with a gradual decline as the rate of tailoring (i.e., personalized content contained within the program) diminished.

## 2. Materials and Methods

### 2.1. Participants

A sample of 339 employees from an Australian transport company were recruited to participate in the study. Participants were aged between 19 and 64 years. Consistent with the organization’s workforce demographics, the majority of participants (62%) were male. Due to operational limitations of the project, including restricted access to employees and work time availability, the number of participants, and volume of program evaluations available, for each program varied considerably. For each program, employees who completed both the program and evaluations are described below.

#### 2.1.1. Tailored Individual Consultation

Nineteen employees (79% male) from three discrete worksites evaluated the tailored individual consultation delivered by a registered dietitian and exercise physiologist. In this program, participant age ranged between 25 and 64 years, with the majority (39%) in the 35 to 44 years cluster.

#### 2.1.2. Semi-Tailored SMS Health Message Program

Two hundred and thirty-four employees (67% male) evaluated the SMS health message program. Participating employees were aged between 19 and 64 years, with the majority (31%) in the 25 to 34 years group.

#### 2.1.3. Standardized Group Workshop

Eighty-six employees (55% male) evaluated the group workshop program. Participants were aged between 19 and 64 years, with most (41%) in the 25 to 34 years cluster.

### 2.2. Design

The study employed a cross-sectional design. Employees’ perceptions of the overall program, affect, and utility for each WHPP were assessed by a combination of quantitative and qualitative items in an evaluation survey.

### 2.3. Procedure

#### 2.3.1. Ethics Approval

This study was approved by the by the Uniting *Care* Health Human Research Ethics Committee (# 2013.12.83) and informed consent was obtained from each participant. All three health programs were designed to promote positive health behaviors aimed at achieving healthy levels of physical activity and nutritional consumption. In all programs, the content was designed to increase participant’s knowledge and motivation, such that employees would be empowered to change or maintain their behavior consistent with the national guidelines [42].

#### 2.3.2. Recruitment

Program trials were advertised by strategically located posters at worksites, promotional emails sent by site Managers to employee email addresses, and in-person promotion during site visits by the research team. During these visits, researchers invited all employees to voluntarily participate in a corporate health survey and trial the WHPP offered at their worksite.

All three trials and evaluation surveys were completed between January and August 2016. In order to maintain the integrity of each trial and comply with the operational requirements of the organization, the specific WHPP offered differed according to geographic site location. Employees were permitted to participate in the WHPP during work hours. To control for fidelity in the delivery of the individually tailored consultation and the standardized group presentation, one expert consultant with qualifications in exercise physiology and dietetics delivered all consultations and workshops. The consultant also contributed to the refinement of the text message content for the SMS health messages program.

#### 2.3.3. Tailored Individual Consultation

Employees were invited to schedule an appointment with the dietitian and exercise physiologist during the trial period. Appointments ranged between 30 and 45 min in duration depending on the specific needs of the participant. The consultation was semi-structured, with the employee self-nominating the nutrition and/or physical activity issues to be discussed. Techniques used by the consultant included: motivational interviewing; goal setting; and the provision of expert nutrition and exercise planning assistance tailored to the individual’s health goals, risks, and current lifestyle behaviors.

#### 2.3.4. Semi-Tailored SMS Health Message Program

Invited employees self-selected to participate in the SMS health message program. The health survey included independent measures of ‘readiness to change’ physical activity and nutritional consumption behaviors based on the readiness ruler used in motivational interviewing theory [43,44]. Participant responses were applied in the process of semi-tailoring the program. Employees who indicated a score of less than or equal to seven out of 10 on the readiness ruler regarding their intention to change their health behaviors were categorized as ‘low readiness to change’. Scores of greater than or equal to eight out of 10, and those who selected ‘*I’m already trying to achieve healthy exercise/eating habits*’ or ‘*I’m already achieving 30 min of moderate exercise on most days/I’m already eating healthy*’, were categorized as ‘moderate to high readiness to change’. Notably, categorization of readiness to change physical activity and nutrition were assessed independently, with many participants being categorized as ‘low readiness to change’ for one behavior (e.g., nutritional consumption) and ‘moderate to high readiness to change’ for the other (e.g., physical activity).

A database of messages was developed by the research team and subjected to an extensive content validity validation process that included a modified Delphi technique with a panel of experts in the fields of health psychology, exercise physiology, and dietetics; and pilot trial with employees of a similar organization [45]. Example SMS messages include: ‘*Fitness is about being a better you. Have you challenged yourself today?*’ (applicable to participants demonstrating a low readiness for exercise health behavior change) or ‘*Read the nutritional panel on your food packaging. For every 100 g, choose items with less than 3 g saturated fat and less than 400 mg sodium*’ (applicable to individuals identified as demonstrating moderate-to-high readiness for nutrition health behavior change).

Participation in the trial required the provision of a mobile telephone number. Participating employees received three text messages per week for a period of 12 weeks with delivery times alternating between 9 a.m. and 4 p.m. local time, on a Monday, Wednesday and Friday.

#### 2.3.5. Standardized Health Workshop

Employees were invited to participate in a 30-minute group workshop on nutrition. The presentation was delivered at five scheduled sessions across two office locations in order to maximize employee participation. The key objective of the workshop was to improve participant understanding of common food behavior patterns and develop positive short-term nutrition intake decisions for long-term health outcomes. Selected content included: the internal thought rationalization process; nutrition for optimal brain functioning; and strategies for improving concentration at work. Employees were provided with the opportunity to ask questions during the session regarding workshop content. No individual or tailored health advice was offered during the workshop sessions.

#### 2.3.6. Program Evaluation

The program evaluation survey was completed by willing participants immediately following the consultation and workshop programs. SMS health message program evaluation surveys were completed during a follow-up site visit at the completion of the 12-week trial.

### 2.4. Measures

#### 2.4.1. Readiness to Change 

**Figure 1:**
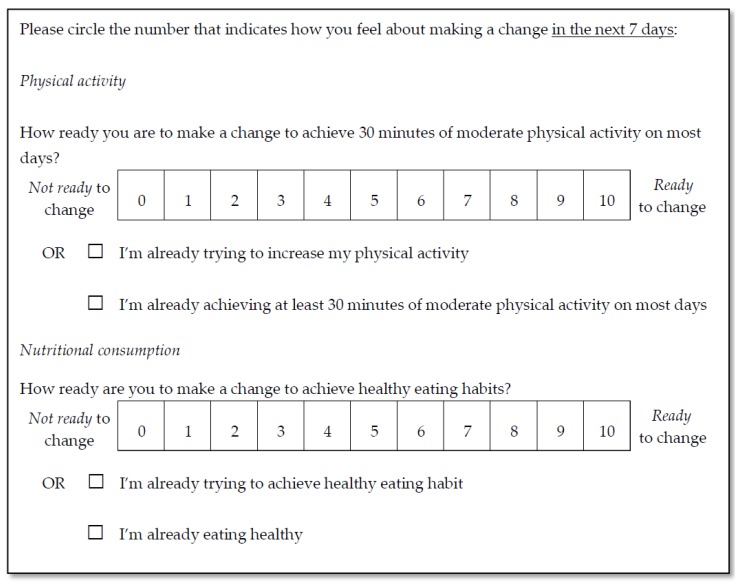
Survey measures of participants’ readiness to change physical activity and nutritional consumption behaviors.

Participants’ readiness to change physical activity and nutritional consumption behaviors were measured as part of the baseline health survey on an 11-point scale, with two supplementary response options indicating active progress toward behavioral change and achievement of national physical activity and nutrition guidelines [42], refer to Figure 1.

#### 2.4.2. Overall Program Rating

Employee perception of the overall program was assessed in the evaluation survey by a single quantitative item followed by a series of three open-response qualitative measures. The quantitative item of overall program rating asked, ‘*Overall, how would you rate the service?*’ and was presented as an 11-point Likert scale with response options ranging from 0 (very poor) to 10 (excellent). The qualitative survey items were based on Walthouwer and colleagues’ [46] evaluation of an obesity prevention intervention. Items included: ‘*What did you like most about the service*?’; ‘*What did you like least about the service?*’; and ‘*Do you have any suggestions to improve the service?*’.

#### 2.4.3. Affect and Utility

**Figure 2:**
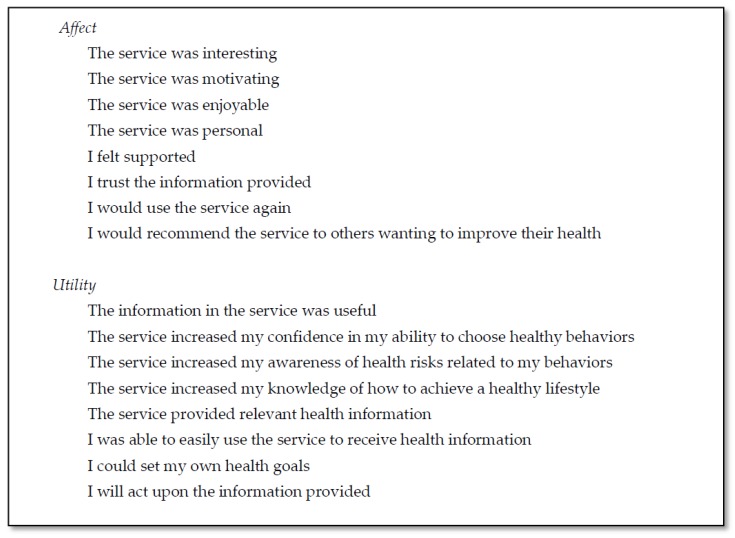
Measures of participants’ perceived affect and utility for each workplace health promotion program.

Measures of participants’ affect and utility of the trials were each assessed in the evaluation survey by eight items measured on a 5-point Likert scale ranging from one (strongly disagree) to five (strongly agree). These items (shown in Figure 2) were based on health promotion program evaluation items developed by Crutzen et al. [20,30].

## 3. Results

Data consisted of participants’ responses to both quantitative and qualitative items in the program evaluation survey. As a result of consistent observations of breaches of normality, all analyses of quantitative data were subjected to a bias-corrected and accelerated (BCa) bootstrap model with 1000 samples using IBM SPSS version 21 [47].

### 3.1. Overall Rating

Table 1 illustrates the descriptive statistics of overall program rating for each program condition. A Kolmogrov–Smirnov distribution analysis revealed that scores were significantly non-normal across all three program conditions, *D* (19) = 0.334, *p* < 0.001; *D* (161) = 0.142, *p* < 0.001; *D* (123) = 0.215, *p* < 0.001, respectively. Levene’s test confirmed that variability in program ratings differed between the groups (*F* (2, 300) = 25.81, *p* < 0.001) with variability in overall rating greatest in the semi-tailored SMS health message program. 

Overall program ratings were high, with the greatest satisfaction being reported in the tailored individual consultation condition. A bootstrapped one-way ANOVA was performed to compare overall program ratings. The ANOVA test showed that differences in program ratings were significant, and the effect of program type on participant rating was large, *F* (2, 300) = 93.31, *p* < 0.001, *η*^2^ = 0.38. Post hoc Dunnett T3 comparisons revealed that the mean overall rating for the semi-tailored SMS health messages program was significantly lower than both the tailored individual consultation (*MD* = −3.055, 95% CI = −3.68, −2.43, *p* < 0.001) and standardized group workshop, *MD* = −2.57, 95% CI = −2.95, −2.20, *p* < 0.001. However, no significant difference between the overall rating of the tailored individual consultation and standardized group workshop was detected, *MD* = 0.49, 95% CI = −0.06, 1.03, *p* = 0.093, n.s.

A thematic analysis was conducted on the qualitative program experience question responses. The overall results of the thematic analysis are presented in Table 2. In total, six themes were identified in the program evaluation feedback, including: authority; content; delivery; self-awareness; tailoring; and other. 

Qualitative feedback on what participants *liked most* (*n* = 210) about the programs revealed that across all three programs, responses related to program content were common (consultations = 31.6%; SMS messages program = 60.4%; group workshops = 44.2%). For the consultation condition, responses that reflected the program tailoring were also common (31.6%) and reflected the personalized nature of the advice provided. Workshop participants also provided frequent responses related to delivery (44.2%) and overwhelmingly reflected the engaging presentation style of the presenter.

Across all program conditions, few participants (*n* = 66) responded to the question regarding what they *liked least* about the program offered. Consultation participants’ responses predominantly aligned with the theme of self-awareness (80.0%) and reflected difficulty related to the process of behavior change (e.g., “*Knowing I have lots of work ahead of me*”). SMS program participant responses to this question varied considerably with the most common responses related to program content (25.9%) and tailoring (25.9%). Content theme responses reflected the generalized nature of message content that was referred to by one respondent as “common sense”; whereas tailoring theme responses reflected participants’ desire for a service more customized to their personal circumstances and health goals. Overwhelmingly, participants reported that what they *liked least* about the program related to delivery (85.3%), and requested the service to be provided on a regular basis and/or for a greater duration.

One hundred and one employees provided *suggestions for improvement* of the workplace health promotion service trailed. Within both the consultation and workshop conditions, responses most frequently related to program delivery (consultation = 83.3% and workshop = 62.2%). For both programs, participants overwhelmingly requested an increase in the frequency and duration of the service. In the SMS program condition, the most common response related to the theme of program content (41.4%), and reflected participants’ desire for more detailed information and resources to supplement the information provided in the SMS messages.

### 3.2. Affect and Utility

Table 3 presents the descriptive statistics relating to affect and utility for each program condition.

#### 3.2.1. Scale Reliability

A reliability test of the affect and utility scales was conducted. Results of the reliability test revealed that the scales of affect and utility both held overall very high internal reliability, *n* = 8, Cronbach’s α = 0.93; *n* = 8, Cronbach’s α = 0.94, respectively. Furthermore, an analysis of the interrelatedness of each item to the overall scale showed that the removal of any singular item from either the affect or utility scales would not significantly improve the internal reliability of the scale.

#### 3.2.2. Affect

Analyses of the affect ratings of each program revealed that participants consistently reported a strong affect in the individually tailored consultation and standardized group workshop trials. By contrast, participants in the semi-tailored SMS health messages program reported a positive, but slightly lower, affect. A Kolmogrov–Smirnov distribution analysis revealed that scores were significantly non-normal across the tailored (*D* (19) = 0.318, *p* < 0.001), semi-tailored (*D* (169) = 0.108, *p* < 0.001) and standardized conditions, *D* (140) = 0.118, *p* < 0.001. Levene’s test confirmed that variability in program ratings differed between the groups (*F* (2, 325) = 34.09, *p* < 0.001) with the greatest variability shown in the semi-tailored SMS health message program rating of affect.

A bootstrapped one-way ANOVA of affect rating revealed significant differences, with program trials having a moderately large effect on perceived affect, *F* (2, 325) = 54.96, *p* < 0.001, *η*^2^ = 0.24. Post hoc Dunnett T3 comparisons identified significant differences between all three program trials. Specifically, the mean affect rating for tailored individual consultations was significantly higher than both the semi-tailored SMS program (*MD* = 1.18, 95% CI = 0.94, 1.40, *p* < 0.001) and standardized group workshop, *MD* = 0.39, 95% CI = 0.18, 0.85, *p* = 0.002. Similarly, the mean affect rating was significantly greater for the standardized workshops when compared with the semi-tailored SMS program, *MD* = 0.79, 95% CI = 0.62, 0.93, *p* < 0.001.

#### 3.2.3. Utility

Overall, participants’ program utility ratings were high in the tailored individual consultation and standardized group workshop trials. Participants in the semi-tailored SMS health messages trial rated the program as marginally lower for utility. A Kolmogrov–Smirnov distribution analysis revealed that scores were significantly non-normal across the tailored (*D* (19) = 0.246, *p* = 0.004), semi-tailored (*D* (169) = 0.122, *p* < 0.001) and standardized trial conditions, *D* (140) = 0.129, *p* < 0.001. Levene’s test confirmed that variability in program ratings differed between the groups (*F* (2, 325) = 42.43, *p* < 0.001), with greatest variability of results observed in the semi-tailored SMS health message program trial.

A bootstrapped one-way ANOVA of utility rating revealed significant differences, with program trial having a moderately large effect on perceived program utility, *F* (2, 325) = 50.98, *p* < 0.001, *η*^2^ = 0.24. Post hoc Dunnett T3 comparisons showed that ratings of utility were significantly lower for the semi-tailored SMS health messages program when compared with both the individually tailored consultation (*MD* = −1.10, 95% CI = −0.86, −1.33, *p* < 0.001) and standardized group workshop, *MD* = −0.84, 95% CI = −1.01, −0.68, *p* < 0.001. No significant difference was observed in the utility rating between the tailored individual consultation and standardized group workshop conditions, *MD* = 0.26, 95% CI = 0.39, 0.46, *p* = 0.084, n.s.

## 4. Discussion

The current study both confirms and extends upon contemporary literature highlighting the importance of employee perceptions in designing and evaluating WHPPs. Specifically, this study was the first to apply the evaluation constructs of affect and utility, as described by Crutzen et al. [20,30], together with an overall program rating [46] to three WHPPs offered within a single organization. With each WHPP offering varying degrees of individual tailoring and mode of delivery, this study demonstrates that employee perceptions of a program’s design substantially contribute to the uptake, likely ongoing participation in, and loyalty to, an offered WHPP. The findings of each evaluation construct are subsequently discussed. 

### 4.1. Overall Rating

Contrary to our prediction, all three programs received moderate-to-high overall ratings which may suggest that employees value efforts by their employer to support worker health and provide WHPP services in general. Notably, higher levels of satisfaction were reported for the WHPPs that offered the tailored individual consultation and standardized workshop conditions when compared to the semi-tailored SMS trial, suggesting that the physical presence of a highly regarded and engaging expert presenter may have positively influenced overall program ratings more than the degree of tailoring provided within the program.

These hypotheses are further supported by the qualitative feedback in which the presentation style and manner of the exercise physiologist and dietitian was overwhelmingly reported as a positive aspect of the program by participants in the consultation and workshop trials. Similarly, SMS program participants were least satisfied with the content and tailoring of the program, despite the researcher’s efforts to match the level of messaging to their reported readiness to change health behaviors. This feedback may also reflect the unidirectional nature of SMS messaging and absence of the physical presence of an expert with whom participants could build a rapport. More generally, this finding may also suggest that personal communication, regardless of whether it be delivered via a private or a group setting, is more highly valued by employees in WHPPs than programs delivered through a virtual platform (e.g., SMS messaging). 

For all three trials, participants most frequently reported program content as what they *liked most* about the service. This reflects previous research which found that employees value WHPPs that promote health education and disseminate relevant information more highly than programs that approach health behavior change via simple give-aways, such as the provision of free fruit in lunch rooms, which may be perceived to be tokenistic [48].

Within the workshop trial condition, employees initially reported 30 minutes as an appropriate session duration that could be completed during their lunch break. Many attendees later requested that longer sessions be offered on an ongoing basis due to the reported perception that additional information would have been appreciated. However, longer workshop session durations may limit participation by those who are unable to extend their lunch break due to work commitments. These requests for longer and ongoing support by participants is supported by the literature suggesting that the frequent contact improved participant outcomes and sustained health-promoting behaviors [6]. Similarly, although some SMS trial participants suggested that the service may be improved by the addition of links to websites containing further information such as recipes, such suggestions may be impractical where such links would, by necessity, be those of external providers. This would also nullify the use of the SMS delivery program—adopted with the 160-character limit such that the content of each message was required to be pointed and concise—as well as potentially compromising the program delivery as the ongoing content of any provided links could not be regulated by the employer. Additionally, providing access to such external resources may substantially increase the cost of program delivery depending on the approach adopted.

### 4.2. Affect

Measures of affect comprise the emotional responses and reactions of program participants and reflect how individuals feel about aspects of the program (e.g., interest, enjoyment, and trust, etc.). On average, overall ratings of affect were high across all three programs. According to Crutzen et al. [30], such high levels of reported affect are associated with high levels of program engagement and would likely result in sustained participation. The hypothesis that the affect ratings would be greatest in the individual tailored condition, with a gradual decline as the rate of tailoring (i.e., personalized content) diminished, was not supported by the findings in this study. While overall affect ratings were highest in the tailored individual consultation condition, ratings were higher for the standardized workshop than the semi-tailored SMS trial. This trend is consistent with that of the overall program ratings and may further infer that the in-person delivery of program content, and presentation style or rapport between the audience and presenter, may lead to more positive perceptions of affect and the program overall, with degree of tailoring playing a lesser role in employee perceptions of program design than previously reported. It is, however, noted that variability of results was also highest in the SMS condition, a finding which may reflect the substantial difference in participation rates or may suggest that the SMS program design and content was more appealing to some employees than others.

### 4.3. Utility

Ratings of program utility represent cognitive responses to the program (e.g., ease of use and program value, etc.). In the present study, the trends observed for measures of affect were replicated in the utility data and are also incongruent with the study hypothesis. In particular, the average overall affect ratings were high across all three trials, which has been associated with elevated user loyalty to the program [41]. Overall utility ratings were highest in the tailored individual consultation and standardized workshop conditions, with no statistical difference observed between the utility ratings of these programs. However, the semi-tailored SMS trial received significantly lower ratings, and contained greater variability in responses. The comparatively lower rating of perceived utility by participants in the SMS trial further indicates that employees prefer the experience of a physical person delivering such programs over that of a unidirectional information service, thereby suggesting that mode of delivery was more influential than degree of tailoring in influencing employee perceptions in this study. It is possible that higher variability in the perceptions of utility in the SMS trial may reflect the substantially higher number of participants in the trial or may also be related to variability in the level of technological literacy of users. This effect could potentially be lessened where employers were able to offer concurrent WHPPs for participants to self-select the mode of delivery they found most appealing. That is, programs that rely on technological platforms (e.g., computer, mobile telephone, or video gaming technology), such as the SMS trial, should be offered alongside more traditional designs (e.g., educational workshops). However, it would be expected that substantially increased costs would be associated with the development and delivery of multiple WHPPs by a single employer, which may render such an approach impractical in many circumstances.

### 4.4. Clinical Implications

This study provides practical contributions to the applied workplace health promotion literature. Based on the findings herein, the employees’ perception of any offered WHPP represents an integral variable which is predicted to translate into observable differences in rate of user engagement (i.e., participation) and program loyalty. Therefore, the practice of WHPP development should be refined to include consideration of employee perceptions and preferences. 

Furthermore, the findings of this study are incongruent with the current scientific literature whereby, unlike the findings of previous studies, program tailoring was not a primary variable in determining the success of the WHPP in terms of user engagement and participation. However, it should be noted that the success of voluntary WHPPs is highly dependent upon rates of participation, and therefore employee perceptions. In this study it was observed that employee perceptions of the overall program, affect, and utility were of a similarly high level for participants in both the tailored and standardized study conditions, with significantly lower ratings in the semi-tailored condition. As the degree of tailoring typically influences program administration costs, this finding indicates that tailoring may play a less important role in attracting participation than previously reported. That is, standardized, more cost-effective programs such as standardized group workshops can be as attractive to employees as the more expensive tailored consultations, as long as the content of the offered WHPP is perceived as favourable by employees. Moreover, qualitative feedback from participating employees implied that mode of delivery, and in particular the physical presence of an expert health practitioner with whom they could build a rapport, was more important to employees than the degree to which the information provided was tailored to the individual. This result was demonstrated regardless of the extent to which the provided contact was individual- or group-based, and further supports the findings that program mode of delivery may also have a greater influence on user engagement and loyalty than program tailoring.

### 4.5. Limitations

There are a number of limitations to the current study that require comment, including: the cross-sectional design; the low response rate for the evaluation survey; sampling bias; replication of the consultation and workshop programs; and differences in program intensity. Firstly, while the cross-sectional design employed in the current study is an important first step toward understanding employee perceptions of different WHPPs, longitudinal studies are needed to investigate whether positive affect and utility ratings translate into participant engagement and loyalty, as predicted. Secondly, the low response rate for evaluation surveys, particularly in the tailored individual consultation condition, represents a major limitation to the current study. Arguably, this reflects the difficulty of obtaining feedback from employees following the conclusion of the free service, primarily due to time and operational constraints. However, regardless of the reason for this low rate of response, such an outcome reduces confidence in the accuracy of any follow-up findings. Thirdly, the recruitment strategy of seeking voluntary participation in WHPPs may have contributed to a selection bias whereby employees self-selecting into the programs disproportionally represent those who are already engaging in healthy lifestyle behaviors rather than those who require the most assistance with health behavior change but are least likely to volunteer for participation. Fourthly, the consultation and workshop programs received extensive positive feedback about the presenter and presentation style, this relates to the individual characteristics of the presenter in this case and evaluation results may vary where a different presenter was employed in a replication study. Finally, it is imperative to acknowledge that this study involved the comparison of WHPPs that differed in intensity (i.e., a 12-week SMS trial versus a one-off consultation or workshop). This notable difference in duration of engagement may have further influenced employee perceptions of the program to an extent greater than that provided in the evaluation survey results. 

Overall, the limitations of this study are consistent with other applied research within the workplace health promotion discipline. However, the authors acknowledge that the project may have been improved by quantifying: (1) the number of employees who declined to participate; (2) WHPP attrition rates; and (3) the rate of conversion from program completion to evaluation.

### 4.6. Future Research

Based on the findings in this study, it is recommended that future research investigate how to improve employee perceptions of WHPPs, particularly for engaging with those least likely to volunteer for participation. Such studies may include the measurement of what motivates employees to participate or which aspects of the program are most appealing to potential participants. Following this, employee feedback and preferences should be investigated and applied in the design and/or refinement of organization-specific WHPPs and evaluated longitudinally to confirm the previous findings that positive affect and utility ratings lead to increased participation [40] and loyalty [41].

## 5. Conclusions

This study compared employee perceptions of overall program rating, affect and utility across three WHPPs. Each offered program had differing levels of tailoring and mode of delivery. Overall, a trend was observed in the employee perceptions which revealed that participant ratings of the overall program, affect, and utility were similarly higher for the tailored individual consultation and standardized workshop condition when compared to the semi-tailored SMS trial. These findings suggest that the physical presence of a highly regarded expert may positively influence the overall program ratings of a standardised WHPP. Based on previous findings, higher ratings of participant perception are predicted to lead to higher levels of user engagement and loyalty within a WHPP. The synergy in ratings between the individually tailored consultation and standardized group workshop indicates that low-cost delivery health programs may, if delivered by a suitable expert, potentially be as appealing and beneficial to employees as comparatively high-cost options. This study was the first to apply the evaluation constructs of affect and utility to workplace health programs and highlights the importance of considering employee preferences in the design of such programs to ensure maximum effectiveness, including employee participation and loyalty.

## Figures and Tables

**Table 1:** Descriptive Statistics for Overall Program Evaluation Scores.

Program	x	*SD*	95% CI
Tailored individual consultations	9.42	0.83	[9.02, 9.82]
Semi-tailored SMS health messages program	6.37	2.07	[6.04, 6.69]
Standardized group workshop	8.93	1.10	[8.74, 9.13]

Note. x = mean, *SD* = standard deviation, 95% CI = 95% confidence interval.

**Table 2:** Thematic Analysis for Overall Program Qualitative Feedback.

Program	Item/Theme	*n*	Example Response
Tailored individual consultations			
	What did you ***like most*** about the service?	**19**	
	Authority	3	*“The doctor knew his stuff”*
	Content	6	*“Information was relevant”*
	Delivery	2	*“Healthy people advising”*
	Other	1	*“Everything!”*
	Tailoring	6	*“Personalized, helpful, informative”*
	Self-awareness	1	*“Cam was able to identify possible health risks in my life I wasn’t aware of that I have been wanting to change”*
	What did you ***like least*** about the service?	**5**	
	Content	1	*“Green tea, hate the stuff!”*
	Self-awareness	4	*“Having to look at how I have let myself down”*
	Do you have any ***suggestions to improve the service***?	**6**	
	Content	1	*“Worthwhile information for an aging workforce”*
	Delivery	5	*“Please provide regularly”*
Semi-tailored SMS health messages program			
	What did you ***like most*** about the service?	**96**	
	Authority	2	*“Factual”*
	Content	58	*“Thought provoking topics”*
	Delivery	16	*“I liked the regular health reminder”*
	Other	4	*“It was a discussion point with work colleagues”*
	Self-awareness	13	*“Simple reminders to stop and think about my own health”*
	Tailoring	2	*“The messages reinforced that I am making good decisions”*
	What did you ***like least*** about the service?	**27**	
	Content	7	*“The advice was very general and ‘common sense’. I didn’t benefit from the information given”*
	Delivery	6	*“Our irregular starting times meant that I was getting SMS messages while sleeping”*
	Other	6	*“It probably works for some, but not for me”*
	Self-awareness	1	*“I need to make more of a commitment”*
	Tailoring	7	*“I felt as though the messages assumed I was unhealthy”*
	Do you have any ***suggestions to improve the service***?	58	
	Authority	4	*“More of the factual messages”*
	Content	24	*“Links to health resources would be good* e.g., *recipes”*
	Delivery	14	*“Reduce the number of messages to 1 or 2 a week”*
	Other	2	*“Ditch it, it has no relevance to me and provided no practical benefits”*
	Tailoring	14	*“Make it more personalized* e.g., *get each person to provide their goals and custom messages to that”*
Standardized group workshop			
	What did you ***like most*** about the service?	**95**	
	Authority	6	*“Strongly evidence based”*
	Content	42	*“Explanation of brain function and fitness”*
	Delivery	42	*“Presenter was engaging, motivating, well-spoken and had great examples”*
	Other	4	*“I feel a bit more motivated”*
	Self-awareness	1	*“Raised my awareness”*
	What did you ***like least*** about the service?	**34**	
	Content	5	*“Didn’t address how people are confused by the multiplicity of often conflicting health messages”*
	Delivery	29	*“Not enough time to discuss, needed an hour”*
	Do you have any ***suggestions to improve the service***?	**37**	
	Content	14	*“More healthy eating suggestions/plans”*
	Delivery	23	*“Regular check in and on-going sessions”*

**Table 3:** Mean Affect and Utility Evaluation Rating of Three Workplace Health Promotion Programs.

	Tailored Individual Consultations			Semi-Tailored SMS Health Messages Program			Standardized Group Workshop		
	x	*SD*	95% CI	x	*SD*	95% CI	x	*SD*	95% CI
**Affect**									
The service was interesting	4.78	0.43	[4.57, 4.99]	3.58	1.16	[3.38, 3.78]	4.64	0.54	[4.54, 4.73]
The service was motivating	4.72	0.56	[4.44, 5.01]	3.58	1.00	[3.40, 3.76]	4.55	0.59	[4.45, 4.64]
The service was enjoyable	4.67	0.69	[4.33, 5.01]	3.44	1.12	[3.25, 3.64]	4.61	0.53	[4.52, 4.70]
The service was personal	4.67	0.49	[4.43, 4.91]	3.46	0.88	[3.30, 3.62]	3.75	0.83	[3.61, 3.89]
I felt supported	4.78	0.43	[4.57, 4.99]	3.47	0.94	[3.30, 3.63]	3.92	0.78	[3.79, 4.05]
I trust the information provided	4.78	0.43	[4.57, 4.99]	3.56	1.26	[3.34, 3.79]	4.52	0.56	[4.42, 4.61]
I would use the service again	4.72	0.58	[4.44, 5.01]	3.71	1.20	[3.49, 3.92]	4.34	0.65	[4.23, 4.44]
I would recommend the service to others wanting to improve their health	4.78	0.43	[4.57, 4.99]	3.64	1.16	[3.44, 3.85]	4.44	0.63	[4.33, 4.54]
Overall affect	4.74	0.43	[4.52, 4.95]	3.54	0.88	[3.39, 3.70]	4.35	0.48	[4.26, 4.43]
**Utility**									
The information in the service was useful	4.67	0.49	[4.43, 4.91]	3.55	1.14	[3.35, 3.75]	4.57	0.53	[4.48, 4.66]
The service increased my confidence in my ability to choose healthy behaviors	4.67	0.49	[4.43, 4.91]	3.47	0.89	[3.31, 3.63]	4.12	0.72	[4.00, 4.25]
The service increased my awareness of health risks related to my behaviors	4.44	0.78	[4.05, 4.83]	3.60	1.13	[3.40, 3.80]	4.26	0.73	[4.14, 4.39]
The service increased my knowledge of how to achieve a healthy lifestyle	4.56	0.62	[4.25, 4.86]	3.63	1.08	[3.44, 3.82]	4.28	0.69	[4.17, 4.40]
The service provided relevant health information	4.72	0.46	[4.49, 4.95]	3.54	1.21	[3.33, 3.75]	4.54	0.65	[4.43, 4.65]
I was able to easily use the service to receive health information	4.56	0.51	[4.30, 4.81]	3.62	1.35	[3.38, 3.86]	4.50	0.58	[4.41, 4.60]
I could set my own health goals	4.67	0.49	[4.43, 4.91]	3.49	0.94	[3.33, 3.66]	4.28	0.63	[4.18, 4.39]
I will act upon the information provided	4.67	0.49	[4.43, 4.91]	3.51	1.01	[3.33, 3.69]	4.31	0.60	[4.21, 4.41]
**Overall utility**	4.62	0.47	[4.39, 4.85]	3.55	0.89	[3.40, 3.71]	4.40	0.49	[4.28, 4.44]

Note: x = mean, *SD* = standard deviation, 95% CI = 95% confidence interval.
